# Superbugs: The Race to Stop an Epidemic

**DOI:** 10.3201/eid2605.191717

**Published:** 2020-05

**Authors:** Andrew F. Read

**Affiliations:** Pennsylvania State University, University Park, Pennsylvania, USA

**Keywords:** antimicrobial resistance, epidemics, bacteria, New York City

Infectious disease professionals should not be put off by this book’s title. Sensationalist titles are de rigueur for books aimed at the general public. I do hope the title grabs that audience because the more lay readers who read this book, the better (great gift for families, friends, and especially young persons seeking meaningful careers). However, this book contains plenty of information for professionals, too. And it is a rattling good read that will greatly shorten a long flight.

Written by Matt McCarthy, a physician-scientist at Weill Cornell Medicine (New York, NY, USA), Superbugs is a vitally important tale of one arena in which the antimicrobial resistance crisis is playing out: New York City (Figure). McCarthy gives the book narrative drive by focusing on a clinical trial of a new antimicrobial drug (dalbavancin). He conducted the drug trial and only recently completed it, so the tale is personal and has a gripping immediacy. Using the trial as a hook, McCarthy discusses many aspects of antimicrobial resistance, from its origins (historic and within patients) to the history of antimicrobial drugs, medical ethics, medical malpractice, health economics, and perhaps most powerful of all the impact of antimicrobial resistance on individual patients and the physicians who care for them. 

**Figure:**
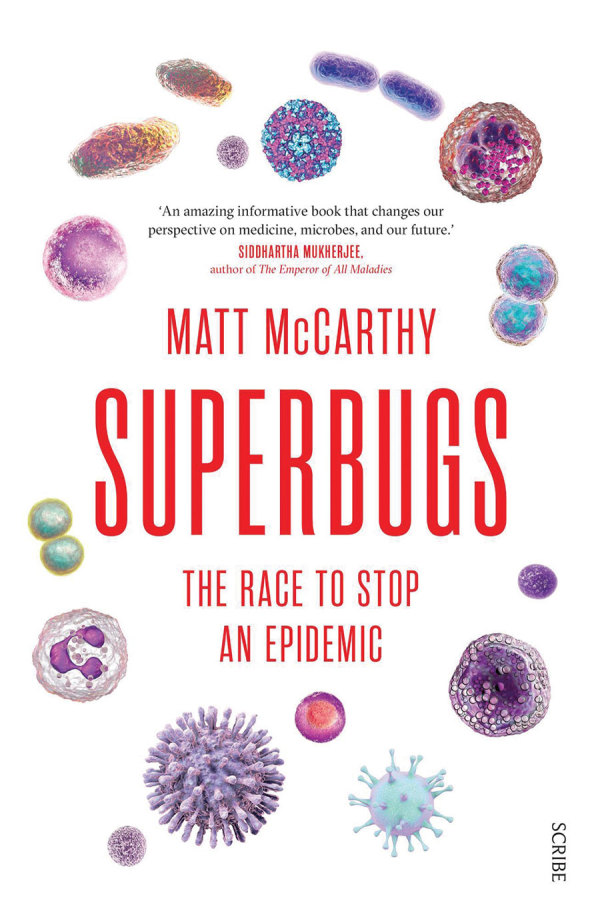
Superbugs: the race to stop an epidemic.

McCarthy is quite the storyteller—he even manages to wrestle drama and excitement out of the institutional review board process—but he is at his best describing the human impact of failing antimicrobial drugs. Healthcare professionals in the trenches with patients will find good therapy here. Professionals, like me, who work on antimicrobial resistance but do not see patients will be reminded why the work is so vital. And professionals who work on other aspects of infectious diseases and wonder whether antimicrobial resistance is overhyped, I implore you to read this important book.

Antimicrobial resistance is a complex, multifaceted problem. No magic bullets will make the problem vanish. It will be with us forever. McCarthy, like many, argues that much use of antimicrobial drugs is inappropriate (for example, for farming or viral infections), and of course we should avoid using them when they are unnecessary. I’d love to understand how much inappropriate use exacerbates the problem created by appropriate use of antimicrobial drugs. I know of no quantitative analysis. It is quite possible that medically appropriate use is the main driver of resistance evolution.

With a drug trial at the heart of the book, McCarthy argues the key solution lies in discovering new drugs. Yet, as he vividly points out, returns on investment are awful for antimicrobial development—and getting worse. Other avenues being explored—vaccines, phages, probiotics, combination therapies—seem as likely to break us out of unsustainable open-ended drug discovery treadmills. Whatever the answer, McCarthy’s book makes a strong case that we can make serious inroads on antimicrobial resistance if we focus scientific and clinical firepower on the problem. His stories of patients show that we really must.

